# From bonding to action: The influence of generalized and interpersonal trust on voluntary membership among European adults

**DOI:** 10.1371/journal.pone.0335260

**Published:** 2025-10-31

**Authors:** Julia Sánchez-García, Marta Gil-Lacruz, Ana Isabel Gil-Lacruz, Miguel Ángel Cañete-Lairla

**Affiliations:** 1 Department of Leadership, University Defense Center of Zaragoza, Zaragoza, Spain; 2 Department of Psychology and Sociology, University of Zaragoza, Zaragoza, Spain; 3 Department of Business Direction and Organization, University of Zaragoza, Zaragoza, Spain; Duke University, UNITED STATES OF AMERICA

## Abstract

This study examines the moderating role of social trust (generalized and particularized/interpersonal) at the national level on the relationship between age (middle-aged 45–59 years; older adults 60–74 years; and long-lived 75 years and older) and membership in voluntary organizations in general and of various types. We hypothesize that in all three age groups, people in countries with higher levels of general and specific trust are more likely to engage in volunteer activities. At the same time, participation in volunteer activities is expected to decline gradually with age. The sample comprises individuals over 45 years of age (*N* = 28,198) in 36 countries in Europe. The empirical estimation uses data from the 2017/22 European Values Survey. Multilevel analysis is used to allow hierarchical aggregation of variables from different levels: individual, national and welfare system. The study reveals that generalized trust is positively associated with volunteering membership among people aged 45 and older. However, it is interpersonal trust that is positively related to voluntary membership among people aged 75 years and older. Furthermore, the influence of the two types of trust varies according to the type of membership. The research highlights that although public and social policies in recent years have promoted the voluntary activity of older adults, not all ages are the same; each age group has a series of characteristics that must be taken into consideration for such an increase in volunteering to take place at all ages.

## Introduction

The literature on the contributions that older people make to communities after retirement as volunteers, providing unpaid services to individuals through formal religious, educational, or other organizations is growing [1,2]. Volunteering strengthens social cohesion by maintaining social contact, as well as promoting social recognition, especially after retirement when the opportunity to be a paid active agent takes a back seat [3,4]. In the last decades, there has been an intensified political and sectoral effort to increase participation in volunteering due to the benefits it generates for the individual and society [5], as it promotes active aging and reduces population aging concerns [6]. Indeed, volunteering rates of older people have increased significantly in the last 20 years compared to younger people [7]. Specifically, Morawski et al. [8] pointed to several countries where volunteering is the most popular (Denmark, Switzerland, and Belgium) and where rates are lowest (Poland, Greece, Czech Republic, and Spain) in European adults aged 50 and above

The literature also shows that voluntary participation is unevenly distributed across older population groups [7,9,10]. For instance, some studies report that people of early retirement age (e.g., ages 60–65) participate in volunteering as much as people who are still of working age until about age 75 or older when a decline in volunteering rates is observed [11]. However, the current underlying dynamics of volunteering among older adults that have promoted this increased participation in certain groups are still not well understood. Some researchers have suggested that increased volunteering participation among older adults could be due to factors such as improved health conditions, higher educational levels, and the promotion of active aging in political discourse [12,13]. Other studies attribute changes in volunteering to differences between specific birth cohorts [14]. Nevertheless, not everything can be due to political discourse as well as improvement in quality of life.

In general terms, knowledge about volunteering by different age ranges or specific groups is still limited. Although there is a considerable number of studies focused on youth and older people, there is much less information on other age groups and on comparisons between them not only in volunteering in general, and especially in different categories of volunteering in recent years [15]. Since the literature assumes that there is an association between different types of social trust and volunteering behavior [16], we questioned whether trust could be one of the factors that facilitate or hinder volunteer activity in older people depending on the age group to which they belong. Given the large cultural differences that could exist between countries in how individuals progress through the years [17], the intersection of life cycle stage and cultural background are important variables in understanding the dynamics of volunteering. This article contributes to a better understanding of the motives that drive volunteering in different age groups currently.

### Volunteering and older adults: Trends in Europe

Lindsey and Mohan [18]define volunteering as “help given, or work done, without remuneration that is of benefit to people beyond one’s immediate family” (pp.6). Specifically, Hoereth [19] defines formal volunteering as volunteering that occurs within a formal organization. Membership in voluntary organizations defined as “....a more formalized and sustained engagement compared to episodic volunteering”. It is associated with institutional ties and a sense of collective belonging” (p. 170) [20]. However, membership in an association does not necessarily mean volunteering, since a person can be a member of a volunteer organization as a donor without taking any action. An active member of an association is a person who regularly provides his or her services to fulfill the group’s objectives [21]. An inactive member, on the other hand, refers to those who do not carry out any activity, but only pay certain dues. Both active and inactive members are considered as associative volunteers. In this study, membership in a voluntary organization is considered without expressly mentioning its activity [22].

Age has been considered an important demographic determinant of volunteering [23]. In this study, we focus on three age groups [24] in middle age (45–59 years), older adults (60–79 years), and long-livers (over 75 years). Throughout the life cycle, individuals adjust their priorities, which impacts decisions about how they allocate their time and energy [25]. Consequently, participation in volunteer activities also varies by life stage, affecting motivations, forms of involvement, and the effects associated with such experiences [26]. However, the literature exploring formal volunteering in different age groups in recent years is limited.

Recent evidence from different countries shows that there are variations in levels of volunteering by age group. Hansen et al. [27] examined the dynamics of volunteering and life satisfaction in midlife and old age in twelve European countries, reporting an increase in volunteering in older adults aged 60 + . According to Strauss [28], from 2004 to 2015, participation in volunteering organizations in people aged 50 + was high in Northern European countries (such as 34% in the Netherlands and 29% in Denmark), while lower in Switzerland (28%), especially Southern and Eastern European countries (such as 5% in Spain, 7% in the Czech Republic and 8% in Estonia). In other English studies including the Community Life Survey, the highest rates of volunteering were found in those aged 50+ [29]. Relatedly, studies have shown that retirement is a period of increased volunteer activity [30,31].

Conversely, there are several studies that have found a negative relationship with age, i.e., reporting that the likelihood of volunteering decreases with aging [32]. Specifically, in the UK [29], they showed that in 2019/20, the volunteer rate was high among the 35–54 age groups, decreasing from age 55–75. In Germany [33], significantly fewer people in the 76 and older age group (12%) were involved in voluntary work than in the 43–75 age groups (20% to 23%) in 2023, which could suggest a ‘peaking’ at certain points in life. Despite these negative findings some suggest that when other sociodemographic factors, individual characteristics [34] or national [15] are considered, volunteering does not necessarily decrease with age. In relation, middle-aged people report volunteering more than older people in political and professional volunteering, while older people in social awareness volunteering [35]. Therefore, it is particularly useful in potentially explaining why volunteering participation could change around life-course transitions.

### Theoretical perspectives on volunteering throughout the life cycle

The life course perspective proposes that people’s actions depend on their personal history and circumstances, such as education and occupation [36,37]. Therefore, performing volunteering may change throughout the life course due to life dynamics, capacity, availability, and motivation [38]. According to resource theory, high levels of human, social and cultural capital promote individuals to invest their time in volunteering [39]. Wilson and Musick [40] explain that volunteering is a type of productive participation that requires human capital (e.g., education, income, time, and health), a collective action that requires social capital (e.g., social connections, trust), and an ethical behavior that requires cultural capital (e.g., connections to religious organizations).

The research on human capital has shown that higher socioeconomic status in terms of income, education, and occupation promotes volunteering by providing the necessary knowledge and opportunities for volunteering [39]. Studies on social capital have found that social connections provide the necessary resources such as information, job sharing, and trust that provide the necessary opportunities to volunteer [39,41]. In relation to marital status, being married provides the social, emotional, and financial support needed to create a broader social network that facilitate access to volunteering than being widowed, divorced, or separated [32]. Social trust comes into play as a social capital. Some studies have found a weak relationship with volunteering [42], while others find a strong relationship [43]. Given the mixed results further research is needed that attends to the conceptualization of social trust, and its role in older volunteering.

Finally, cultural capital seeks a culture of “benevolence” that precipitates volunteering [44]. Much of the literature has focused on religion as an accumulation of this social capital that promotes helping others, especially as it relates to religious volunteering [5,39]. However, not all volunteering is the same, as there are others such as social awareness (e.g., humanitarian issues), political and professional (e.g., trade unions and political parties), and educational and leisure time (e.g., sport or art) [35,45]; nor can religion be the sole cultural promoter, as European welfare systems can be [46,47].

The welfare systems investigated in the literature refer to the Anglo-Saxon, continental, Nordic, Mediterranean and eastern countries, which can be divided into central and peripheral systems [48–51]. The social origins theory of welfare systems explains the probability of voluntary participation in terms of the contextual factors that determine it, such as economic development [52]. Authors Gil-Lacruz et al. [53] through the European Values Survey found that welfare systems influence government spending and employment influence youth volunteering decisions. Regarding older age, Sánchez-García et al. [35] reported that people over the age of 50 in Nordic countries report volunteering more overall and in every volunteering category than residents of continental, Mediterranean and eastern countries; except for volunteering of a religious nature. Thus, welfare systems play a key role in the multidimensional study of volunteering among older people.

### Generalized and particularized trust as correlates of volunteering in an aging population

Previous studies have focused on conceptual models characterizing various forms of social trust [54,55]: generalized and particularized/interpersonal trust [16,56] to examine the relationship between social trust and volunteering in older people. Generalized trust (trusting in general across the world) [55] promotes social activities that help strangers, as it guides individuals toward common goals and to pursue them collectively [57]. A large body of research shows that people with a high level of trust tend to have higher civic engagement than those with lower general trust [16,43,58,59].

Particularized/interpersonal trust “extends only to people the individual knows from everyday interactions” (p. 784) [54]. According to some studies, this type of trust does not promote volunteering by distinguishing between “us” and “them” among the citizenry [16,60]. However, previous studies focus on general or political volunteering [61], and thus it is unknown what happens in other categories of volunteering as well as in each age group at present.

Okun and Schultz [62] found that older adults were more motivated than younger adults to participate in volunteering activities for the purpose of strengthening social relationships, denoting the importance of considering social trust in studies of the relationship between age and volunteering. Nevertheless, hardly any research is found on the relationship between age and social trust [63–65], and only two that consider such a relationship in volunteering or civic engagement [61,66].

Sutter and Kocher [65] used the trust game to assess trust in different age groups. No age differences were found, however. On the other hand, Robinson and Jackson [64] noted that older adults tend to have higher generalized trust than younger adults. Li and Fung [63] reported that age differences in particularized trust (e.g., family or friends) were smaller than generalized trust (e.g., strangers) in 38 countries around the world. The authors report that age is positively related to generalized and interpersonal trust.

Regarding its relationship with volunteering, Botzen [58] points out a bidirectional relationship between generalized trust and volunteering, the effect of trust on volunteering being stronger than the other way around. In addition, Jennings and Stoker [66] indicate that trust is a cause rather than a consequence of civic participation and that the interdependence between social trust and volunteering is more evident as people age, with a decline in trust and volunteering found in Generation X and not in Baby Boomer. Sønderskov [67] analyzes the WVS and finds that generalized trust increases membership in groups, although this relation is found only for associations producing public goods. Pavlova and Lühr [61], noted a more positive relationship of general volunteering and generalized trust in younger adults than in older adults, and that this varied across European countries. Liu et al. [16] report an inverse relationship between interpersonal trust and volunteering. However, they did not examine whether this varied as a function of age group.

Salamon and Sokolowski [68] pointed out that the microstructural approach to volunteering focused on social capital does not provide a good explanation for differences in volunteering membership across countries. Furthermore, they point out that it is necessary to consider macro-structural explanations to understand differences in volunteering across countries, as these helps explain patterns of individual behavior at the national level. However, the literature so far has assessed social trust from an individual rather than a multidimensional approach, so that variations in volunteering across countries by age may depend on the type of social trust that prevails in each region.

### The present research

In order to adequately capture the relationship between age and volunteer participation, the variable was recoded into three groups (middle age, young older adults, and long-livers). Several studies have shown that the association between age and volunteering is not linear, but curvilinear: participation peaks in middle age and tends to decline progressively in later stages of the life cycle [13,26,69–71];. Categorization into age groups therefore allows for a clearer interpretation and facilitates comparison with previous literature that has identified this pattern.

Specifically, in the present study, we investigated cross-national differences in the links between three age groups (middle-old, ages 45–59 years; older adults, 60–74 years; and long-livers, 75 + years) and membership in general volunteer organizations, and in three typologies: 1) Social awareness and social justice; 2) Professional and political; 3) Educational and leisure. We also examine the moderating role of social trust of two types in the above relationships across 36 European countries from the European Values Survey (EVS; European Values Survey Round 5 Data, 2017–2022). Specifically, we studied generalized trust and particularized/interpersonal trust. Based on previous studies on volunteering and psychosocial development throughout the life cycle, the following hypotheses are put forward:

H1. Membership in volunteer organizations differs significantly among age groups (middle-old, older adults, and long-livers).

H2. People with higher levels of generalized social trust are more likely to be members of voluntary organizations, whereas particularized trust is negatively associated with membership in voluntary organizations.

Moreover, considering the role of social trust as a possible moderator:

H3. Generalized trust and particularized trust moderate the relationship between age and membership in associations, such that the negative effect of advanced age on membership is less pronounced among those with higher levels of trust.

## Methods

### Ethics statement

This study is based on secondary data from the European Values Survey (EVS), a cross-national longitudinal survey that examines social and individual factors related to membership in voluntary organizations. The fifth round of the EVS (2017–2022) was selected because it provides the most recent and comprehensive data available on individuals’ social values, membership in voluntary groups, and trust, which were downloaded on February 4, 2025, from the official EVS repository (https://europeanvaluesstudy.eu).The link to access the database is https://osf.io/smbqy/overview?view_only=a261710740fc43b89d929069c6c35066

The data used are fully anonymized and publicly available for research purposes. The original data collection was conducted by EVS and its national partner organizations, which obtained informed consent from participants and complied with relevant ethical and legal requirements in each country. The authors of this study did not have access to participants’ identifying information at any time. Because the authors did not directly collect data or come into contact with human participants, no additional ethical approval was required for this secondary analysis.

### Data

The final sample consisted of 28.198 individuals aged over 45 years. The sample was selected to be able to make comparisons between three age groups: middle adult (40–59 years), older adult (60–74 years), and senior citizen and long-lived older adult (75 years and over).

To measure participation in voluntary organizations as dependent variable, we used several questions from the EVS questionnaire that ask whether the respondent mentions membership in different types of organizations. From these variables, three dichotomous indicators grouped thematically were generated. Each takes the value 1 if membership in at least one organization in the group is mentioned, and 0 if none is mentioned. Following previous studies on volunteerism [45], we aggregated membership in a volunteering organization in general and into three groups: (1) Membership in social awareness and social justice organizations (membersocialawareness): Religious organizations or associations, conservation organizations, the environment, ecology, animal rights, humanitarian or charitable organization, self-help group, mutual aid group; (2) Membership in political or professional organizations (memberprofessionalpolitical): political parties, labor unions, professional associations, consumer groups; (3) Membership in educational or recreational organizations (membereducation): Sports or recreations, education, arts, music or cultural activities. It is worth mentioning that in our data; only one organization fell into the “social justice” category (religious associations). For this reason, and following previous evidence that the determinants of volunteering in religious organizations and other social awareness organizations point in the same direction, we added them into a single category, “social awareness and social justice organizations” [15].

The gender variable was assessed through the response to the question about the respondent’s gender. The variable has the following categories: 1 = Male: Response corresponding to male respondents and 2 = Female: Response corresponding to female respondents. For operationalization, this variable was kept in its binary form (male/female). The income variable was measured through the respondents’ income scale position, where they are asked to place their household on a scale from 1 to 11, where 1 = Lowest step (worst economic situation) to 11 = Highest step (best economic situation). For operationalization, this scale was grouped into three categories: Low income: steps 1–4; Medium income: steps 5–7 and High income: steps 8–11. Educational level was classified using the International Standard Classification of Education [72]. The EVS recodes the responses into a three-level ordinal variable: Low studies: ISCED 0–2 (primary or lower secondary education); Middle studies: ISCED 3–4 (upper secondary and post-secondary non-tertiary); High studies: ISCED 5–6 (tertiary education). Marital status was measured through the question on the respondent’s marital status. The variable was classified into the following categories: Married: People who are legally married and living together as married; Divorced: People who are divorced or separated; Widowed: People who are widowed; and, Single: People who are unmarried or never married.

The key national variables we introduced in this study are related to different notions of trust. To measure generalized trust [26], the question “Would you say that most people can be trusted or that you cannot be too careful in dealing with others?” was used. Responses were coded as 1 for generalized trust and 0 otherwise. To measure interpersonal trust in close relationships, the item “I trust people I know personally” was used, measured on a Likert-type scale from 1 (I trust completely) to 4 (I do not trust at all). In order to maintain consistency with the generalized trust variable, the interpersonal trust variable was recoded by assigning the value 1 to those who selected the highest levels of trust (1 = “I trust completely” and 2 = “I trust somewhat”), and 0 to those who selected lower levels of trust (3 = “I do not trust much” and 4 = “I do not trust at all”).

Following Hardin’s [73] distinction, it is important to distinguish between trust and trustworthiness. On the one hand, trust refers to people’s beliefs about the reliability of others and is therefore a subjective perception. On the other hand, trustworthiness refers to the actual characteristics of people or the social environment that make them worthy of such trust and is therefore an objective property of the context. In this study, interpersonal and generalized trust at the individual level reflects respondents’ personal expectations of others, while trust of both types at the national level, calculated as the average trust in a country, can be interpreted as a contextual indicator that partially reflects the structural trustworthiness of the environment. Including both measures in the models allows us to differentiate between compositional effects—how each individual’s beliefs about trust influence membership in volunteer organizations—and contextual effects—how a society’s climate of trust influences membership in a volunteer organization regardless of individual beliefs.

To classify countries according to their social welfare systems, six categories were created based on the classic welfare models proposed by Esping-Andersen [74]. The categories are: (1) Nordic welfare system: Sweden, Denmark, Finland, Norway and Iceland; (2) Anglo-Saxon welfare system: Great Britain; (3) Continental welfare system: Germany, Netherlands, France, Austria and Switzerland; (4) Mediterranean welfare system: Spain, Italy and Portugal; (5) Central Eastern welfare system: Poland, Hungary, Czech Republic, Slovakia, Slovenia, Estonia, Latvia, Lithuania and Croatia; (6) Eastern Peripheral Welfare System: Bulgaria, Romania, Serbia, Bosnia and Herzegovina, Montenegro, North Macedonia, Albania, Russia, Belarus, Ukraine, Armenia, Georgia and Azerbaijan. The welfare system categories were operationalized as dichotomous variables (0 = Does not belong to this welfare system, 1 = Belongs to this welfare system).

### Empirical framework

The empirical strategy was based on multilevel models (STATA: melogit), given the hierarchical structure of data (individual, national, and welfare systems), with a dependent variable measured at the lowest level and a set of independent variables on each of the levels. The response variable was a dichotomous measure of being a member. The advantage of multilevel regression models is that they use methods such as fixed effects and stronger controls for omitted variable bias [75]. To show parameters indicating both the meaning and intensity of the effects of explanatory variables the results of the estimated coefficients were summarized in tables. In this study, data are structured for the individual (i=1,…, 28,198 ) of j-countries (j=1,…, 36) through a nonlinear response model. Thus, the dependent variable, ${Member}_{ij}$, was estimated as:


$${Membership}_{ij}=X_{ij}^{\prime }\beta +u_{j}+e_{ij}$$ (1)

in which $X_{ij}$ represents the set of independent variables and involves the appearance of K  regressors (K−1 variables and a constant),  β are the regression coefficients common to all countries, $u_{j}$ is a random intercept that captures heterogeneity across countries, and $e_{ij}\approx N(\text{0},\;\sigma^{\text{2}})$ as the error term. The challenge consisted of estimating β as accurately as possible. In contrast to country-by-country estimation, our β are not country-specific; however, in contrast to pooled OLS estimation, inclusion of the random effects accounts for the variation in the basic probability of membership across countries while not precluding the simultaneous inclusion of country-level variables. The analyses were repeated three times for each type of voluntary organization (f=1 membersocialawareness; f=2  memberprofessionalpolitical; f=3  membereducation).


$${Membership}_{fij}=X_{ij}^{\prime }\beta_{f}+u_{fj}+e_{fij}$$ (2)

To separate the individual and contextual effects of trust, we estimated multilevel models that simultaneously included interpersonal and generalized trust variables at both the individual and national levels. Four models were estimated: Model 1 included only individual-level trust variables; Model 2 included only national-level trust variables; Model 3 included both levels simultaneously; and Model 4 included interactions between national trust and various age groups, in addition to welfare systems as a macro-level control variable. *Pseudo‐R*^*2*^ was calculated following Snijders and Bosker’s [76] method to assess model fit, reaching 77% for general membership, 71% for membership in social awareness and social justice organizations, 86% for membership in professional and political organizations, and 81% for membership in education and leisure organizations in Model 3, showing a high fit through the inclusion of explanatory variables.

To test whether performing a multilevel analysis was appropriate, we checked the intraclass correlation index. In the null model, that is, without predictors for ${Membership}_{ij}$, the intraclass correlation coefficient (ICC) was 0.43 for general membership, indicating that approximately 43% of the variance in membership in voluntary organizations was explained by cross-country differences. For membership in social awareness associations, professional, and political organizations, it was 49%, while for membership in education and leisure organizations, it was 62%. This justified the use of a multilevel model, as a considerable proportion of the variance cannot be attributed solely to individual factors (Huang, 2018).

## Results

### Preliminary analysis

Before estimating the multilevel models, the possible collinearity between the independent variables was examined by calculating the Variance Inflation Factor (VIF) in a simple logistic model without random effects. All VIF values were below the commonly accepted threshold of 10, suggesting the absence of significant collinearity between predictors [77]. This preliminary check supports the validity of the specification of the multilevel models subsequently employed.

### Descriptive and correlational analysis

Descriptive statistics are presented below for the main variables and broken down by age group: Middle adult (40–59 years), older adult (60–74 years), and long-lived older adult (75 years and over) noting means and standard deviations (Table 1.). The level of higher education and upper income was higher for individuals aged 45–59 years (*M* = .36, *SD* = .48; *M* = .17, *SD* = .36) than for those aged 60–74 years (*M* = .29, *SD* = .45; *M* = .09, *SD* = .29) and 75 years and over (*M* = .23, *SD* = .42; *M* = .04, *SD* = 20). In general, scores for membership in volunteering organizations were medium (*M* = .50, *SD* = .50). Specifically, membership in political and professional organizations varied by age group, with the mean being highest for the 45–59 years middle-aged group (*M* = .30, *SD* = .46), compared to the older group (*M* = .22, *SD* = .41), senior and long-lived group (*M* = .15, *SD* = .36). Regarding trust, interpersonal trust showed high scores overall (*M* = .89, *SD* = .31), while moderate for generalized trust (*M* = .38, *SD* = .49).

**Table 1:** First descriptive analysis.

	Total		45-59		60-74		75+	
	Mean	SD	Mean	SD	Mean	SD	Mean	SD
***Gender***								
Female	.56	.50	.55	.50	.56	.50	.58	.49
Male	.44	.50	.45	.50	.44	.50	.42	.49
***Education Level***								
Lower studies	.23	.42	.16	.37	.26	.44	.42	.49
Middle studies	.45	.50	.48	.50	.46	.50	.34	.47
Upper studies	.31	.46	.36	.48	.29	.45	.23	.42
***Income Level***								
Low income	.51	.50	.40	.49	.56	.50	.72	.45
Middle income	.37	.48	.43	.49	.35	.48	.24	.43
High income	.12	.32	.17	.36	.09	.29	.04	.20
***Marital Status***								
Married	.61	.49	.67	.47	.62	.48	.42	.49
Single	.08	.28	.12	.33	.06	.23	.04	.19
Divorced	.14	.34	.17	.37	.13	.33	.08	.27
Widow	.17	.37	.05	.21	.19	.39	.46	.50
***Employment status***								
Employed	.43	.50	.75	.43	.22	.42	.04	.19
Retired	.43	.50	.05	.22	.68	.47	.90	.30
Unemployment	.10	.31	.15	.36	.07	.26	.05	.21
***Voluntary membership***								
Memberngo	.50	.50	.52	.50	.50	.50	.46	.50
MemberSocialAwareness	.31	.46	.30	.45	.32	.47	.32	.47
MemberPolitical&Professional	.25	.43	.30	.46	.22	.41	.15	.36
MemberEducation&Leisure	.25	.43	.26	.44	.25	.43	.20	.40
***Individual trust***								
Interpersonal trust	.89	.31	.89	.31	.90	.30	.90	.30
Generalized trust	.38	.49	.39	.49	.38	.49	.37	.48
***Welfare system***								
Nordic	.17	.38	.17	37	.18	.38	.16	.36
Anglo-Saxon	.04	.19	.06	.18	.03	.18	.04	.21
Continental	.21	.41	.20	.40	.20	.40	.23	.42
Central Eastern	.22	.41	.19	.39	.23	.42	.24	.43
Eastern Peripheral	.31	.46	.35	.48	.30	.46	.25	.43
Mediterranean	.05	.23	.06	.23	.05	.22	.07	.26

*Note*. Variables indicate types of organizational membership: Memberngo (general), MemberSocialAwareness (social awareness), MemberPolitical&Professional (political/professional), MemberEducation&Leisure (educational/leisure).

Correlation analyses were then performed to assess the associations between the main study variables. interpersonal and generalized trust at national level showed a positive and significant correlation with membership in general organizations (*r* = .41, *p* < .001; *r* = .48, *p* < .001), membership in social awareness and social justice organizations (**r* *= .35, *p* < . 001; **r* *= .42, *p* < .001), political and professional organizations (*r* = .30, *p* < .001; *r* = .38, *p* < .001), and education and leisure (*r* = .35, *p* < .001; *r* = .38, *p* < .001), indicating that trust of both types are positively associated with voluntary membership.

The middle-aged group showed a negative association with interpersonal trust at the national level (**r* *= −.03, **p* *< .001), whereas the older (*r* = .02, *p* < .001) and senior and long-lived (*r* = .02, *p* < .001) groups showed a positive association with it. These results should be interpreted with caution due to the different levels of data aggregation at the individual and national levels.

### Multilevel analysis

Table 2 shows the aggregate estimates of the probability of being a member of voluntary organizations in general. In Model 1, the results showed that people aged 60–74 were positively associated with membership in volunteer organizations in general. Having high education level, high income, being married, and being employed were positively associated with such membership. No significant differences were found for gender. The significant variance of the random effect, *σ²,* supported the use of a multilevel model to analyze voluntary membership. Interpersonal and generalized individual trust was positively associated with membership in voluntary organizations.

**Table 2:** Multilevel Logistic Regression Estimates for Volunteering Organization Membership.

	Model 1	Model 2	Model 3	Model 4
***Regression coefficients***				
Female	0.06	0.06	0.06	0.05
Maleª	--	--	--	--
Age1: 45–59ª	--	--	--	--
Age2: 60–74	0.11**	0.13**	0.11*	−0.80
Age3: 75+	0.07	0.09	0.07	−1.80**
Lower studiesª	--	--	--	--
Middle studies	0.15***	0.18***	0.15***	0.15***
Upper studies	0.78***	0.85***	0.78***	0.78***
Low incomeª	--	--	--	--
Medium income	0.26***	0.28***	0.26***	0.26***
High income	0.35***	0.40***	0.35***	0.37***
Marriedª	--	--	--	--
Single	−0.06	−0.06	−0.06	−0.05
Divorced	−0.15***	−0.17***	−0.16***	−0.15***
Widow	−0.02	−0.03	−0.02	−0.01
Employed	0.42***	0.43***	0.42***	0.42***
Retired	0.05	0.06	0.05	0.04
Unemployedª	--	--	--	--
Individual Interpersonal trust	0.26***	--	0.26***	0.26***
Individual Generalized trust	0.40***	--	0.40***	0.40***
National Interpersonal trust	--	2.11	1.97	−3.05
National Generalized trust	--	4.42***	4.11***	1.21
Trust*Age group				
Interpersonal trust*Age2	--	--	--	1.00
Interpersonal trust*Age3	--	--	--	2.07**
Generalized trust*Age2	--	--	--	0.10
Generalized trust*Age3	--	--	--	0.06
Nordicª	--	--	--	--
Anglo-Saxon	--	--	--	−1.13
Continental	--	--	--	−0.77
Central Eastern	--	--	--	−1.93**
Eastern Peripheral	--	--	--	−2.61***
Mediterranean	--	--	--	−2.38***
***Random effects***				
σ2	1.41	0.49	0.50	0.35
LR test (Prob > χ^2^)	0.00	0.00	0.00	0.00
ICC	0.30	0.13	0.13	0.10
*Pseudo‐R*^*2*^	0.30	0.70	0.70	0.77

*Note.* The “Regression coefficients” show the regression coefficients (β) that are common to all countries. The “Random effects” reflect the random intercepts per country (u_j_), which capture the variability between countries.

^*a*^Variable of reference.*** *p* < 0.001, ** *p* < 0.05, * *p* < 0.01.

In Model 2, it was shown that national generalized trust was positively related to overall voluntary membership. The unexplained variance was reduced (*σ² = *.49, *p* < .001) respect to Model 1 (*σ²* = 1.41, *p* < .001) with the introduction of the national-level measures. In Model 3, the results showed that generalized trust and interpersonal trust at the individual level remained significant predictors with the introduction of national measures. Generalized trust at the national level was also significant when included alongside individual measures. In contrast, interpersonal trust at the national level ceased to be significant when controlling for trust at the individual level. In Model 4, the interaction term between national interpersonal trust and the oldest age group (75+) was positive and statistically significant. This suggests that, in contexts where higher interpersonal trust prevails, older people are more likely to be members of voluntary organizations, compared to their counterparts in lower trust settings, without assuming causality. The interaction term for generalized trust was not significant. Furthermore, residing in a Nordic country was positively associated with overall voluntary membership compared to residing in Eastern and Mediterranean countries.

Table 3 shows the aggregate estimates of the probability of reporting membership to a voluntary organization related to social awareness and social justice. In Model 1, being a woman was positively associated with this type of voluntary membership. No significant results were found regarding employment status. In terms of age, the second (60–74 years) and third age groups (75+) showed a positive relationship with this category of voluntary membership. Both types of trust were positively related to membership in social justice voluntary organizations. In Model 2, national generalized trust positively increased the likelihood of belonging to social awareness and social justice voluntary organizations. In Model 3, with the simultaneous introduction of individual and national measures, all types of trust maintain their level of significance, with interpersonal trust at the national level being the only one that is not significant. Finally, in Model 4, the interaction between national generalized trust and the second (60–74 years) and third age group (75+) was found to be significant. Thus, in contexts where generalized trust is higher, people over 60 are more likely to belong to social awareness volunteer organizations.

**Table 3:** Estimations for volunteering organization membership: Social Awareness organizations.

	Model 1	Model 2	Model 3	Model 4
***Regression coefficients***				
Female	0.40***	0.40***	0.40***	0.40***
Maleª	--	--	--	--
Age1: 45–59ª	--	--	--	--
Age2: 60–74	0.14***	0.14***	0.13***	0.78
Age3: 75+	0.17***	0.20***	0.17***	0.02
Lower studiesª	--	--	--	--
Middle studies	0.03	0.11**	0.08**	0.05
Upper studies	0.36***	0.45***	0.38***	0.36***
Low incomeª	--	--	--	--
Medium income	0.07*	0.08**	0.06	0.07
High income	0.13**	0.15***	0.11**	0.12**
Marriedª	--	--	--	--
Single	−0.05	−0.08	−0.07	−0.06
Divorced	−0.20***	−0.22***	−0.21***	−0.20***
Widow	−0.01	−0.02	−0.01	−0.01
Employed	−0.00	0.01	−0.01	−0.01
Retired	0.03	0.03	0.02	0.01
Unemployedª	--	--	--	--
Individual Interpersonal trust	0.15**	--	0.13**	0.14**
Individual Generalized trust	0.45***	--	0.40***	0.40***
National Interpersonal trust	--	0.49	0.40	0.74
National Generalized trust	--	3.77***	3.41***	2.97***
Trust*Age group	--	--	--	--
Interpersonal trust*Age2	--	--	--	−0.92
Interpersonal trust*Age3	--	--	--	−0.29
Generalized trust*Age2	--	--	--	0.46*
Generalized trust*Age3	--	--	--	0.96***
Nordicª	--	--	--	--
Anglo-Saxon	--	--	--	−0.52
Continental	--	--	--	−0.36***
Central Eastern	--	--	--	−1.56***
Eastern Peripheral	--	--	--	−2.98***
Mediterranean	--	--	--	−2.73***
***Random effects***				
σ2	4.11	1.04	1.06	0.72
LR test (Prob > χ^2^)	0.00	0.00	0.00	0.00
ICC	0.56	0.24	0.24	0.18
*Pseudo‐R*^*2*^	0.05	0.59	0.59	0.70

*Note.* The “Regression coefficients” show the regression coefficients (β) that are common to all countries. The “Random effects” reflect the random intercepts per country (u_j_), which capture the variability between countries.

^*a*^Variable of reference.*** *p* < 0.001, ** *p* < 0.05, * *p* < 0.01.

Table 4 replicates the same procedure for membership in professional and political voluntary organizations. In Model 1, the results indicated that being male was positively associated with membership in professional and political organizations. Being employed was positively associated with membership in these types of organizations. As in the previous case, both types of trust at the individual level were positively related to membership in professional and political organizations. In Model 2, national generalized trust was positively related to the probability of belonging to these types of voluntary organizations. In Model 3, national interpersonal trust was once again not significant. In Model 4, in contexts of high interpersonal trust, the probability of being a member of political and professional organizations was higher in individuals aged 60 and older. However, in contexts with high generalized trust, the probability of being a member was reduced in individuals aged 60 and older compared to the group aged 45–59 years.

**Table 4:** Estimations for volunteering organization membership: Professional & political organizations.

	Model 1	Model 2	Model 3	Model 4
***Regression coefficients***				
Female	−0.20***	−0.20***	−0.21***	−0.21***
Maleª	--	--	--	--
Age1: 45–59ª	--	--	--	--
Age2. 60–74	0.04	0.05	0.04	−1.69***
Age3: 75+	−0.04	−0.03	−0.03	−4.36***
Lower studiesª	--	--	--	--
Middle studies	0.25***	0.28***	0.25***	0.25***
Upper studies	0.87***	0.92***	0.87***	0.87***
Low incomeª	--	--	--	--
Medium income	0.32***	0.33***	0.31***	0.32***
High income	0.33***	0.34***	0.31***	0.31***
Marriedª	--	--	--	--
Single	−0.06	−0.08	−0.07	−0.04
Divorced	−0.04	−0.05	−0.04	−0.00
Widow	−0.12**	−0.13**	−0.12**	−0.12**
Employed	0.87***	0.88***	0.86***	0.86***
Retired	−0.02	−0.03	−0.05	−0.05
Unemployedª	--	--	--	--
Individual Interpersonal trust	0.37***	--	0.36***	0.36***
Individual Generalized trust	0.28***	--	0.25***	0.25***
National Interpersonal trust	--	−0.09	−0.50	−1.27
National Generalized trust	--	3.79***	3.61***	2.87***
Trust*Age group	--	--	--	--
Interpersonal trust*Age2	--	--	--	2.32**
Interpersonal trust*Age3	--	--	--	5.47***
Generalized trust*Age2	--	--	--	−0.84**
Generalized trust*Age3	--	--	--	−1.59***
Nordicª	--	--	--	--
Anglo-Saxon	--	--	--	−0.37
Continental	--	--	--	−0.21
Central Eastern	--	--	--	−0.67
Eastern Peripheral	--	--	--	−0.52
Mediterranean	--	--	--	−0.95
***Random effects***				
σ2	2.23	0.29	0.29	0.27
LR test (Prob > χ^2^)	0.00	0.00	0.00	0.00
ICC	0.40	0.08	0.08	0.07
*Pseudo‐R*^*2*^	0.18	0.84	0.84	0.86

*Note.* The “Regression coefficients” show the regression coefficients (β) that are common to all countries. The “Random effects” reflect the random intercepts per country (u_j_), which capture the variability between countries.

^*a*^Variable of reference.*** *p* < 0.001, ** *p* < 0.05, * *p* < 0.01.

Table 5 replicates the same procedure for membership in educational and leisure volunteer organizations. In Model 2, national interpersonal trust was positively associated with membership in educational and recreational organizations, while national generalized trust showed a negative association. This indicates that, in contexts where greater interpersonal trust predominates, people tend to be more linked to educational and leisure volunteer organizations; in contrast, in contexts with greater generalized trust, this form of membership is less frequent. In Model 3, being older than 75 years was negatively associated with this type of volunteer membership. Model 3 showed that in contexts with high generalized trust, people over 75 years of age are more likely to participate in this type of voluntary organization. In contexts with high interpersonal trust, people between the ages of 50 and 74 are more likely to be members.

**Table 5:** Estimations for volunteering organization membership: Education & leisure organizations.

	Model 1	Model 2	Model 3	Model 4
***Regression coefficients***				
Female	−0.02	−0.01	−0.02	−0.02
Maleª	--	--	--	--
Age1: 45–59ª	--	--	--	--
Age2: 60–74	0.05	0.06	0.04	2.29***
Age3: 75+	−0.10	−0.08	−0.01	−1.16
Lower studiesª	--	--	--	--
Middle studies	0.36***	0.40***	0.38***	0.37***
Upper studies	0.91***	0.99***	0.92***	0.92***
Low incomeª	--	--	--	--
Medium income	0.27***	0.27***	0.26***	0.28***
High income	0.42***	0.45***	0.40***	0.46***
Marriedª	--	--	--	--
Single	−0.06	−0.07	−0.06	−0.04
Divorced	−0.12**	−0.13**	−0.12**	−0.12**
Widow	−0.11	−0.12*	−0.11*	−0.07
Employed	0.51***	0.54***	0.52***	0.53***
Retired	0.40***	0.42***	0.41***	0.42***
Unemployedª	--	--	--	--
Individual Interpersonal trust	0.29***	--	0.28	0.28***
Individual Generalized trust	0.34***	--	0.34	0.33***
National Interpersonal trust	--	5.64***	5.48***	−1.01
National Generalized trust	--	−1.64***	−1.95***	1.10*
Interpersonal trust*Age group	--	--	--	--
Interpersonal trust*Age2	--	--	--	2.58***
Interpersonal trust*Age3	--	--	--	0.59
Generalized trust*Age2	--	--	--	−0.12
Generalized trust*Age3	--	--	--	1.03**
Nordicª	--	--	--	--
Anglo-Saxon	--	--	--	−0.13
Continental	--	--	--	0.42***
Central Eastern	--	--	--	−1.06***
Eastern Peripheral	--	--	--	−1.72***
Mediterranean	--	--	--	−1.00
***Random effects***				
σ2	4.32	1.73	1.75	0.45
LR test (Prob > χ^2^)	0.00	0.00	0.00	0.00
ICC	0.57	0.34	0.34	0.12
*Pseudo‐R*^*2*^	0.08	0.45	0.45	0.81

*Note.* The “Regression coefficients” show the regression coefficients (β) that are common to all countries. The “Random effects” reflect the random intercepts per country (u_j_), which capture the variability between countries.

^*a*^Variable of reference.*** *p* < 0.001, ** *p* < 0.05, * *p* < 0.01.

In addition, robustness tests were performed using the Wald test to assess whether all interactions, linear and quadratic, are simultaneously different from zero, providing direct evidence on whether the relationship between trust and volunteering varies with age. The Wald test of linear and quadratic interactions between age and interpersonal trust (χ²(2) = 5.97, **p* *= .05) indicates that the relationship between national interpersonal trust and general volunteering may vary by age; specifically in professional and political organizations (χ²(2) = 15.74, *p* < .001), and educational organizations (χ²(2) = 11.13, **p* *< .01), except for social awareness volunteering (χ²(2) = 2.02, **p* *= .364). The joint test of interactions between age and generalized trust was not significant in the general volunteering model (χ²(2) =.13, *p* = .94), suggesting that, on average, there is no evidence that the association between trust and volunteering varies with age. However, when analyzing specific types of volunteering, significant interactions are detected in the categories (social awareness, χ²(2) = 8.60, *p* = .01; political and professional, χ²(2) = 17.23, *p* < .001; educational and leisure, χ²(2) = 9.18, *p* = .01), indicating that the relationship between generalized trust and volunteering may depend on the type of organization in question. By grouping all forms of volunteering into a single indicator, these variations are masked and the average effect disappears.

Finally, analyses of variance (ANOVA) were performed for Model 1 for each voluntary membership. Analysis of variance indicated significant differences between groups as a function of the welfare system (General Volunteering: *F*(5, 28193) = 24385.62, *p* < .001, *η²* = . 81; Social Awareness: *F*(5, 28193) = 14508.44, *p* < .001, *η²* = .72; Professional and Political: *F*(5, 28193) = 12979.96, *p* < .001, *η²* = .70; Educational and Leisure: *F*(5, 28193) = 22835.34, *p* < .001, *η²* = .80). This suggests that a substantial proportion of the variance in membership in voluntary organizations can be attributed to differences between countries grouped according to their welfare regimes. In other words, differences in voluntary membership are greater in countries with different regimes than in those belonging to the same regime. This justified the introduction of welfare regimes as a macro-social variable in Model 3.

## Discussion

The main objective of this research was to test whether there were age differences in three groups of adults (middle-old, ages 45–59 years; older adults, 60–74 years; and long-livers, ≥ 75 years) in membership in a voluntary organization in general and in various categories of volunteering in recent years. Moreover, we examined whether the above relationships were moderated by social trust (generalized and particularized/interpersonal). First, the results suggested that being aged 60–74 is positively associated with membership in volunteer organizations in general, in contrast to the younger age group, which is consistent with hypothesis 1. Previous empirical evidence on volunteering in general [32,78,79] explains a higher proportion of volunteering in middle age as a function of individual characteristics, such as higher educational level and income, resources, and health [27,80] as human capital. However, the results found are in line with recent empirical research [2,7]. As an example, Kelle et al. [2] indicate that in Germany, the peak of volunteering seems to be at age 65, with lower volunteering rates at ages below and above 65. In addition, the authors suggest stability in the contributions of volunteer time and effort dedicated by those over 65 compared to earlier cohorts. The increase in volunteering among adults of this age may be due to factors such as improved subjective well-being, higher educational levels, and the promotion of active aging in political discourse [13,51]. The resource theory explains volunteering by high levels of human, social, and cultural capital that enable individuals to invest their time in the volunteer role [81].

The results show that there are age differences according to the type of volunteer membership. Older adults aged 60 and even 75 years report being more likely than the middle age group to belong to social awareness organizations. This is consistent with previous studies showing that self-expressive values focused on progress and humanitarian issues such as equality can promote volunteering by those over 65 in certain organizations such as social awareness organizations aligned with the above values [15].

Second, the results show that both interpersonal and generalized trust at the individual level predict a higher probability of belonging to volunteer organizations (compositional effect). However, at the national level, only generalized trust has an independent contextual effect: even people with low generalized trust at the individual level are more likely to participate in volunteering if they live in countries with high levels of generalized trust. The national effect of interpersonal trust disappears when controlling for individual interpersonal trust, indicating that it was a compositional effect. Specifically, the results reveal that generalized trust is positively associated with membership in volunteering organizations in general in line with Hypothesis 2, and of any type, except for educational and leisure volunteering. This is in line with the results of Glanville et al. [82] who showed that levels of contextual generalized trust are positively related to volunteering because social capital exists at different levels of the social structure, such as groups and communities. Others indicate that it can be due to the role of welfare institutions and states [46] that foster such trust by generating values such as respect for human rights and equal opportunities among others.

The reason why generalized trust is negatively associated with membership in educational and leisure volunteer organizations may be that, in this type of volunteering, interpersonal trust is more important. In relation to this, the findings of Van der Meer and Van Ingen [83] indicate that leisure and sports organizations have the least social capital effects compared to political and social organizations, understanding social capital as generalized trust. However, interpersonal trust is characterized by trusting other people who are known personally, such as family members, relatives, and friends [84].

The fact that national interpersonal trust seems to be positively related to education and leisure volunteering, while generalized trust is related to social, political, and professional volunteering, can be explained by Gordon and Babchuk’s [85] classic typology of associations. According to this theory, there are inward-oriented “ingroup” associations, which pursue the purpose of “serving members” [86] and are oriented “towards providing activities for members as an end in itself” (p. 25) [85]. On the other hand, outward-oriented “outgroup” associations are seen as means through which social or political goals can be achieved, while exerting influence on the social environment. In this sense, it seems that interpersonal trust promotes trust in ingroup members, while generalized trust promotes trust in both ingroup and outgroup members. In relation to this, Burrmann et al. [87] found that members of sports clubs showed less trust in the outgroup, which is in line with this explanation.

Educational and leisure volunteering focuses specifically on activities related to sports, art, and leisure, which are often shared in an environment with people you know. Given this, it seems logical to think that in contexts where trust in people from the close “ingroup” circle is promoted, individuals will join educational and leisure volunteer organizations where such interactions with people they know take place; while in contexts that promote trust in all people, whether or not they belong to the ingroup, this context will be associated with volunteering in political, professional, and socially conscious organizations

Third, the results do not support the hypothesis that generalized trust moderates the relationship between age and membership in associations, which contradicts initial expectations. However, when in a society generalized trust is high, the relationship between older (60–74 years) and long-lived (75+) people and social awareness volunteering is positive, as it is for educational and leisure volunteering for the latter age group. This can be explained by the study of Li and Fung [63], who explain the generalized high trust in older adults based on the socioemotional selectivity theory [88]. That is, older adults may prioritize emotional connection with people in general as a consequence of a limited time perspective, which may lead them to engage in generative activities [62] in contexts where they feel safe. Related to the latter, in contexts of high generalized trust, more structural opportunities and lower barriers to engage in volunteering are found among older people [89], with organizations being more accessible, safe, and inclusive [90], which seems to be more possible in social awareness and educational organizations.

The assumption that, in societies with high generalized trust, middle-aged adults (45–59) volunteer more than those over 60 in organizations of a political and professional nature may be because middle age is the stage of greater professional stability and accumulated human capital in terms of education and work experience, as well as broader social networks [5]. In this regard, Musick and Wilson [26] argue that political and professional volunteering is more likely among people with higher socioeconomic status and trust in others in general.

Regarding interpersonal trust, the findings showed that societies with high interpersonal trust promote volunteering among citizens over 75 years of age. Thus, Hypothesis 3 is partially fulfilled. One possible explanation is that trust toward closer people is generally higher throughout adulthood compared to trust toward strangers [63], so it is possible that older adults’ increasing dependence on their neighborhood [91] is what gives rise to volunteer activity. Specifically, the results show that, at age 60 and older, interpersonal trust is positively associated with participation in political organizations. At older ages, people rely more on their personal and community networks to access opportunities for participation [5], including political ones. This could indicate that, at this stage of the life cycle, face-to-face interaction (e.g., neighborhood movements; acquaintance campaigns or local initiatives) can reinforce political participation through the activation of personal ties.

Finally, in general, and by type of organization, the results informed a positive influence of the Nordic welfare system on the frequency of membership in voluntary organizations. Thus, residents of Nordic countries are more frequently members than residents of continental, Mediterranean and eastern countries. This is aligned with previous literature for youth and adult volunteering [15,53] who explain such a result by the higher gross domestic product in the Nordic countries in contrast to the rest. It can also be explained because in countries with greater equality in terms of income, formal volunteering is promoted [92]. For his part, Enjolras [46] explains the higher volunteering ratios in these countries following the capability approach, pointing to the availability of individual and collective resources (capital) as a promoter of volunteering. The author also notes that the macro-structural indicators of democracy (civil liberties) and horizontal social capital (social trust) are positively associated with volunteering rates, demonstrating once again the importance of social trust examined not only at the individual level but also at the macro-social level.

The public and social policy implications of trust and voluntary membership are significant. Trust is one of the foundations of social capital; thus, the greater the trust in others, as well as in institutions, the greater the likelihood that citizens of any age group will associate with volunteer organizations. In the first place, public policies could focus their efforts on fostering environments of trust through transparency, accountability, and promotion of values. In other words, associations could make it clear in awareness campaigns that some of their objectives are aimed at meeting the needs of their own members (ingroup), while others are aimed at meeting the needs of individuals in society in general (also outgroup). In this way, a context of both interpersonal and generalized trust can be promoted to encourage the inclusion of older people in volunteer organizations of any kind.

Second, they could invest in civic and emotional education to foster social trust from an early age. The literature highlights that social trust—both interpersonal and generalized—has its roots in early life experiences. Erikson’s [93] first stage of psychosocial development (trust vs. mistrust) indicates that the reliability of the caregiver in childhood leads to interpersonal security in adulthood. In addition, longitudinal studies in early stages show that trust during childhood predicts prosocial behavior in later years [94], and that schooling and peer support can foster the development of social trust [95]. Taken together, this evidence indicates that early intervention in family and school contexts may be key to consolidating lasting levels of trust in most people, which has broad implications for the design of social cohesion and volunteerism policies.

Thirdly, public and social policies should consider the virtuous circle of trust and volunteering, i.e., social trust increases volunteering, which in turn promotes social cohesion by increasing social trust. At this point, programs that address the benefits of volunteering at any age [96] could examine what kind of confidence each type of volunteer organization promotes in different age groups. For example, widespread trust seems to be positively related to socially conscious volunteering among people aged 60 and over, but does it also generate it consequently? Finally, reports and programs that are intended to lead to the formulation of public and social policies that promote volunteering [97] should consider that not all trust is equal, and even less so if we disaggregate by age group. Therefore, efforts to promote volunteer activity based on trust differ according to the type of association and age.

### Strengths and limitations

This empirical study consisted of analyses conducted on microdata and macrodata organized from the European Values Survey (2017/22). Including 36 European countries allowed for robust cross-national comparisons and exploration of the effect of socioeconomic, cultural or institutional contexts. Likewise, it enabled analysis by welfare systems (e.g., Nordic, continental, Mediterranean, etc.). The last round allowed us to explore the new dynamics of volunteering among older people in contemporary contexts. The data are open access, well documented and accessible through https://europeanvaluesstudy.eu/ or GESIS, with harmonized questionnaires and available code. However, the study is not without limitations. The fact that the data round is between 2017 and 2022 implies that updated data were collected post-economic crisis and during the pandemic period, offering a unique look at recent societal changes but which may be influencing the results obtained. Nevertheless, the study is not without limitations.

First, various studies indicate that the relationship between social trust and participation in volunteering can be bidirectional. On the one hand, social trust can encourage participation in voluntary associations [42,97]. On the other hand, there is evidence that active participation in volunteering can, in turn, increase trust in others and strengthen social capital [98,99]. In this study, we follow the commonly proposed direction of causality—from social trust to voluntary participation—but given that the data are cross-sectional, it is not possible to establish definitive causal relationships.

Trust variables could introduce technical endogeneity problems, for example, that the variables determining membership in a voluntary organization also conditioned the type of trust [58]. Both effect and causality can produce the relationship between voluntary membership and types of trust. On the one hand, the effect of trust on voluntary membership refers to the fact that trusting individuals may be more likely to join voluntary organizations. On the other, causality refers to the socialization effect of voluntary membership on trust. Future work could use panel data to resolve endogeneity issues and clarify the direction between variables in older individuals. Nevertheless, this paper overcomes the above limitation by examining generalized and particularized trust at the macro level.

Second, this study contemplates membership in a voluntary organization. An active member of an association is a person who regularly provides services to fulfill the group’s objectives [21]. In contrast, inactive members, also known as nominal or “paper” members, are those who do not carry out any activities but only pay certain fees. Both active and inactive members are considered membership volunteers. In this study, we focus on volunteer membership, thus it is not possible to clearly distinguish between active and inactive members. Future studies could focus exclusively on the EVS variable centered on active/inactive volunteering rather than membership, although this variable is rarely collected in the latest waves for European countries. Nevertheless, recent research shows similar results in active volunteering in terms of age differences distinguishing between pre-retirement and post-retirement age [15].

Third, comparisons between welfare systems should be made with caution with respect to the Anglo-Saxon welfare system, since it covers only one country. Future studies could also look at other country groups worldwide, including African-Islamic, Confucian or Latin American, to broaden the understanding of volunteer membership in terms of value-based cultural differences between countries following the modernization theory [100].

### Future research

Given that the objective of this study is to analyze patterns of voluntary participation in middle age and old age, young adults (18–45 years old) were excluded. The literature has pointed out that participation in volunteering during these early stages of life is more conditioned by transitional factors, such as completing studies, entering the labor market, or starting a family, which makes patterns of involvement more unstable and heterogeneous [101]. In contrast, from middle age onwards, volunteer participation stabilizes and more clearly shows the curvilinear pattern documented in previous studies, with a peak around middle age followed by a gradual decline [26,69,71]. However, future research could examine other age ranges to fully clarify whether the relationship between age and volunteering is curvilinear, as well as include other control variables such as employment status.

This study is a good starting point for future research on the life trajectory of volunteering as a function of social trust. Across 36 countries in Europe, we found a pattern whereby age 45 and older was positively related to volunteering with a higher level of generalized trust at the national level. However, when we differentiate between adult age groups, for those over 75 to engage in volunteering in general requires a context with higher interpersonal trust. Thus, social trust (generalized and particularized/interpersonal) operates differently depending on the age group. However, there are also other types of trust (neighbors, family and friends) [63] that could improve the understanding of the relationship between age and volunteering in various categories.

Future studies could examine other types of variables that promote participation in volunteering among older people. For example, participation in religious organizations may be related to individual religiosity, therefore future studies could include it as a control variable. Other socio-cultural and macro-social factors could be investigated in the aging context across countries. Economic factors have been recognized as promoters of social capital in society [102]. In Europe, several cross-national studies have been conducted on the relationship between income inequality and membership of voluntary organizations. For example, Saunders [103] finds that voluntary organization membership has no significant relationship with income inequality, while Lancee and Van de Werfhorst [104] report a relationship between civic participation and absolute income in more unequal countries. However, their volunteering measures only include activities in political and professional organizations and recreational associations, without considering other related ones such as education, religion, or social awareness [105].

Explanations related to economic resources suggest that higher gross domestic product (GDP) or higher government social expenditures could facilitate voluntary participation [47]. The “exclusion” thesis, however, postulates that government welfare expenditure might reduce the likelihood of volunteering in an organization [68]. Relatedly, Steinber [106] and Brooks [107] suggest that each welfare system is characterized by different government expenditures that could have a positive or negative relationship with voluntary organization membership. For instance, the Nordic system is characterized by high levels of government expenditure and high volunteering rates, while post-communist countries show low levels of both. Continental countries combine high levels of government expenditure and medium volunteering rates. Finally, Mediterranean countries show lower levels of government expenditure and volunteering than Continental countries [47]. Thus, European welfare systems are similar, but they also exhibit significant differences. Future studies could examine the influence of GDP and income inequality on the relationships examined in this study.
